# Evaluating Factors Explaining U.S. Consumers’ Behavioral Intentions toward Irradiated Ground Beef

**DOI:** 10.3390/foods12173146

**Published:** 2023-08-22

**Authors:** Jean A. Parrella, Holli R. Leggette, Peng Lu, Gary Wingenbach, Matt Baker, Elsa Murano

**Affiliations:** 1 Department of Agricultural, Leadership, and Community Education, Virginia Tech, Blacksburg, VA 24060, USA; 2 Department of Agricultural Leadership, Education, and Communications, Texas A & M University, College Station, TX 77843, USA; holli.leggette@ag.tamu.edu (H.R.L.); gary.wingenbach@ag.tamu.edu (G.W.); mathew.baker@ag.tamu.edu (M.B.); 3 Department of Agricultural Leadership, Education and Communication, University of Georgia, Athens, GA 30602, USA; penglu@uga.edu; 4 Department of Food Science and Technology, Texas A & M University, College Station, TX 77843, USA; elsa.murano@ag.tamu.edu

**Keywords:** attitudes, communication, consumer acceptance, food irradiation, food safety, food technology neophobia, risk perception, purchase intention, structural equation modeling, social norms

## Abstract

Although food irradiation is deemed safe and endorsed by health-related organizations worldwide, consumers are reluctant to accept the technology. Yet, consumer acceptance is critical as food irradiation has significant potential for increasing the safety and availability of food globally. To communicate about food irradiation, science communicators should understand the psychology behind consumers’ decision making related to irradiated foods. Using empirical research, we developed a theoretical model and used structural equation modeling to determine how nine variables affect consumers’ behavioral intentions toward irradiated ground beef. We purchased a national quota sample from Qualtrics and surveyed *N* = 1102 U.S. consumers. The model explained 60.3% of the variance in consumers’ attitudes toward food irradiation and 55.4% of their behavioral intentions toward irradiated ground beef. Attitude had the largest positive, total effect on consumers’ behavioral intentions, which was followed by subjective social norm and perceived benefit. Perceived risk had the largest negative, total effect on behavioral intentions. Attitude mediated the effect of subjective social norm, perceived benefit, perceived risk, objective knowledge, and food technology neophobia. Environmental concern and health consciousness did not significantly affect behavioral intention. Science communicators should develop messaging strategies that seek to improve consumer acceptance with these factors in mind.

## 1. Introduction

Irradiation, “a safe, healthy, and clean technology,” is a non-chemical food processing and preservation method [1] (p. 91) developed more than 100 years ago [2]. During the energy-efficient irradiation process, ionizing radiation beams (i.e., gamma rays, electron beams, X-rays) are exposed to and sterilize food before it is packaged [1,3]. As a result, the radiation beams “eliminate pathogenic microorganisms, insects, fungi, and pests” [1] (p. 91). Thus, two primary advantages to irradiation exist: increased food safety and food preservation (i.e., shelf life) [1,4]. Irradiating food can reduce consumers’ risk of contracting foodborne diseases and reduce food loss due to spoilage or bacterial/parasitic contamination [1].

Food irradiation was first approved by the U.S. Food and Drug Administration (FDA) to treat foods in 1963 [5]. However, the FDA did not give “permission for the expanded use of irradiation in the U.S. food supply” until 1987 [2]. The FDA approved a variety of foods for irradiation, including beef, pork, poultry, crustaceans (e.g., lobster, shrimp, crab), fresh fruits and vegetables, seeds (e.g., alfalfa), eggs, shellfish (e.g., oysters, clams, mussels, scallops), spices, and seasonings [6]. To this end, about 97 million pounds of food consumed annually in the U.S. is irradiated [7]. Of this, between 15 and 18 million pounds of irradiated food marketed in the U.S. annually is ground beef and poultry [8]. Although many grocery stores in Midwestern states sell these products, nationwide availability is still very limited [7,9].

Even though food irradiation is deemed safe and endorsed by health-related organizations worldwide, consumers are still reluctant to accept the technology [10,11]. As a result, food irradiation has failed to achieve widespread adoption [12]. The reason consumers’ acceptance of food irradiation and its adoption is so important is because the technology has significant implications for strengthening the food supply [13]. Irradiation can increase the safety of the food supply by decreasing the prevalence of foodborne disease—a leading cause of disease and mortality across the globe—and also help secure the global food supply by preserving food [13,14]. Liu et al. [15] found approximately one-third of food produced is thrown out or spoiled prior to human consumption. Such post-harvest losses are the leading causes of hunger and malnutrition [14]. Treating food with irradiation can increase the shelf life of food, sometimes by twofold, thereby increasing food availability [13].

Still, food irradiation has a bad reputation. Like consumers do with other food and agricultural technologies, they tend to evaluate the risks of irradiation differently from experts [16]. Risks that consumers commonly associate with food irradiation involve safety, health, and the environment. Consumers generally believe food irradiation is a dangerous, nuclear technology that modifies food properties and contaminates food, making it unsafe to eat [16]. Much of the fear stems from the word ‘irradiation’ itself because consumers associate it with radiotherapy and radiation, both of which are closely associated with cancer [17]. The belief also exists that irradiation facilities create hazardous environments for workers [18,19]. In addition, many consumers believe the irradiation process decreases the nutritional value of food and negatively affects its color, taste, smell, and texture, even though such effects are minimal [20,21]. Some also fear that radiation escapes from irradiation facilities and pollutes the environment [18,19].

Irradiated foods are also widely unaccepted because the technology was ineffectively diffused into society [11] and because information from food processors about food irradiation has often been communicated poorly [16]. Many scholars have called for better public education and communication about the benefits of food irradiation [22,23] to increase consumers’ acceptance. Due to the ineffectiveness of diffusion and communication efforts, Sapp and Downing-Matibag [11] suggested scholars broaden their theoretical perspective and not simply examine consumer acceptance of food irradiation as a function of perceived risk. A broader empirical perspective may reveal better ways to communicate and educate consumers about food irradiation.

Thus, the purpose of the study described herein was to determine how key factors explain consumers’ behavioral intentions toward irradiated ground beef, specifically, because ground beef is one of the most common U.S. household food products. We achieved the study’s purpose by reviewing the literature to identify key factors that have been found to, or are expected to, influence consumers’ acceptance of food irradiation. We integrated the identified factors to develop a comprehensive theoretical model and evaluated the proposed model using structural equation modeling. Finally, we discussed potential communication strategies focusing on the factors we identified as most significant.

## 2. Theoretical Background and Hypothesis Development

### 2.1. Knowledge

Evidence suggests that consumers’ reluctance to accept food irradiation stems from their lack of knowledge and understanding [16,18,24,25,26,27,28]. Product availability increases consumers’ self-reported knowledge of irradiation technology, and because irradiated foods are largely unavailable, it is not surprising that their knowledge is generally low [29]. Many studies have also found that it is possible to increase consumers’ knowledge about food irradiation by presenting them with factual information about the technology, even if the information provided is minimal, e.g., [30,31,32]. The more consumers know about food irradiation, the more their interest in purchasing irradiated foods increases [30,31,32] and the more positive their attitudes toward irradiation become [29]. Because consumers generally have low knowledge about food irradiation, their judgements toward the technology are based more on affective processes [24]. For example, without knowledge, consumers experience fear and doubt toward the use of food irradiation [27]. Therefore, based on the aforementioned empirical results, we propose the following hypothesis.

**Hypothesis** **1** **(H1).** *Objective knowledge has a significant positive effect on attitude toward food irradiation.*

### 2.2. Environmental Concern

Consumers seek food believed to be environmentally friendly [33]. Many scholars have identified environmental concern as a factor driving food choice behaviors, including those involving genetic engineering, e.g., [18,34,35,36,37,38]. Specifically, high environmental concern is associated with negative attitudes toward genetically modified foods [18]. In the context of food irradiation, consumers often believe the food irradiation process pollutes the environment [18,19,39]. Therefore, environmental risk has been identified as a perception influencing consumers’ judgements about the technology, but the influence of general environmental concern has not been explored. When consumers value the environment and feel concerned for its well-being, they tend to select foods that they believe were produced sustainably using environmentally friendly methods [33]. They also tend to assume that food technologies do not promote environmental sustainability [33]. Because of consumers’ newfound awareness of the environment and interest in sustainability, further investigation is needed to determine how environmental concern affects consumers’ attitudes toward food irradiation. Environmental concern, as a value, may directly affect attitude [40]. Thus, we propose the following hypothesis.

**Hypothesis** **2** **(H2).** *Environmental concern has a significant negative effect on attitude toward food irradiation.*

### 2.3. Food Technology Neophobia

Food technology neophobia is “a personality trait that affects consumers’ willingness to accept new food technologies” [41] (p. 29). Many scholars have identified food technology neophobia as a significant factor explaining consumers’ attitudes toward food technology applications, e.g., [42,43,44]. Specifically, the more food technology neophobic consumers are, the more negative their attitudes are toward food technologies, like nanotechnology and cell-based biotechnology [42,43,44]. No studies that we found have reported results investigating the effect of food technology neophobia on consumers’ attitudes toward food irradiation. This could be, in part, because Cox and Evans developed the food technology neophobia scale in 2008 [45], and many studies investigating consumer acceptance of food irradiation were conducted before 2008, e.g., [46,47]. However, evidence suggests “food technology neophobia is a universal factor that influences acceptance of innovations related to food” [48] (p. 345). Therefore, some scholars have discussed food technology neophobia as a factor that may influence consumers’ acceptance of food irradiation [44,48,49]. But there is a need for concrete evidence documenting this assumed relationship. We propose the following hypothesis.

**Hypothesis** **3** **(H3).** *Food technology neophobia has a significant negative effect on attitude toward food irradiation.*

### 2.4. Health Consciousness

Health consciousness is a sustained trend among consumers [50]. Health-conscious consumers are more aware of their food’s origin and prefer food that they believe is natural (e.g., not processed) because they think it is healthier [51]. We found no studies investigating the influence of health consciousness on consumers’ acceptance of food irradiation. The relationship is worth investigating in our study because various other studies have documented how being health-conscious affects consumers’ acceptance of other novel foods and food technologies, including functional foods [52], high-pressure processing [53], edible insects [54], genetically modified foods [55], and organic foods [51]. When it comes to foods that are generally perceived as natural or healthy, like organic foods or functional foods, health consciousness has a positive effect on attitudes [51,52], but when it comes to foods that are generally perceived as unnatural or unhealthy, like genetically modified foods, health consciousness has a negative effect on attitudes [55]. Health consciousness, as a value, may directly affect attitude toward food irradiation because irradiated foods are often perceived as unnatural and unhealthy [16,17,20,21,40]. Thus, we propose the following hypothesis.

**Hypothesis** **4** **(H4).** *Health consciousness has a significant negative effect on attitude toward food irradiation.*

### 2.5. Perceived Risks and Benefits

Consumers tend to associate food irradiation with less benefits and more risks [56], which is part of the reason they are not accepting of irradiated foods [23,24]. There are various risks consumers commonly associate with food irradiation. Some fear that irradiated food, or its packaging, may be radioactive [1,9,11,17] and associate it with cancer or birth defects [11,17,27]. Some also fear that irradiation unfavorably changes the taste, texture, smell, and nutritional value of foods [11,20,21,57]. Others fear that the irradiation process poses a health risk to employees [18,19,34] and pollutes the environment [18,19]. Previous studies reported that perceived risks influence consumers’ attitudes toward food irradiation [58] and their use of irradiated foods [25]. In terms of benefits, [56] found that perceived benefits influence consumers’ attitudes toward food irradiation. Often, consumers believe irradiation benefits food manufacturers and retailers more than it benefits consumers themselves [18,59]. For example, two important benefits of food irradiation are increased shelf-life and improved food safety, but there is no demand for increased shelf-life from consumers, and although consumers value food safety, they expect their food to be safe [59]. Therefore, the perceived personal benefits of the technology are low [18,59]. Often, consumer acceptance of food irradiation is investigated as a function of perceived risk [11]. Thus, we propose the following three hypotheses.

**Hypothesis** **5** **(H5).** *Perceived benefit has a significant positive effect on attitude toward food irradiation.*

**Hypothesis** **6** **(H6).** *Perceived risk has a significant negative effect on attitude toward food irradiation.*

**Hypothesis** **7** **(H7).** *Perceived risk has a significant negative effect on behavioral intention toward irradiated ground beef.*

### 2.6. Subjective Social Norms

Subjective social norms refer to social influences on actions toward a behavior [60]. They are the result of normative beliefs concerning pressure to conform to actions that are socially appropriate [61]. Subjective social norms influence consumers’ food choices and their acceptance of new food technologies [62]. The novelty of food technologies accompanied by the lack of knowledge leads consumers to rely on the opinions and behaviors of those important to them to aid in their decision making [62]. Although there is limited evidence regarding the role of social norms in consumers’ acceptance of irradiated foods [63], specifically, evidence that does exist is contradictory. For example, [64] found that interactions with family and other social groups influence consumers’ decisions to purchase irradiated foods, and [63] similarly found that social norms in favor of irradiated foods directly influence consumers’ intentions to accept such products. However, [65] found subjective norms did not affect consumers’ intent to consume irradiated foods. Therefore, further investigation regarding the role of social norms is warranted to inform science communication strategies effectively [66]. We propose two hypotheses.

**Hypothesis** **8** **(H8).** *Subjective social norm has a significant positive effect on attitude toward food irradiation.*

**Hypothesis** **9** **(H9).** *Subjective social norm has a significant positive effect on behavioral intention toward irradiated ground beef.*

### 2.7. Attitude

Attitude toward food irradiation is an important factor explaining consumers’ acceptance. For the purpose of the study, we defined attitudes as positive or negative beliefs about food irradiation [29]. Rozekhi et al. [67] found that Malaysian consumers had positive attitudes toward food irradiation and were willing to accept irradiated foods. Teisl et al. [29] found the opposite—U.S. consumers had negative attitudes toward food irradiation. When studying Australian consumers, [63] found that attitudes had a direct, positive effect on their intent to accept irradiated foods, and Buczkowska et al. [68] found the same thing when studying Polish consumers. Prominent theories of behavior change posit that attitudes directly affect behavior [40] and behavioral intention [60]. Thus, we propose the following hypothesis.

**Hypothesis** **10** **(H10).** *Attitude has a significant positive effect on behavioral intention toward irradiated ground beef.*

**Figure 1:**
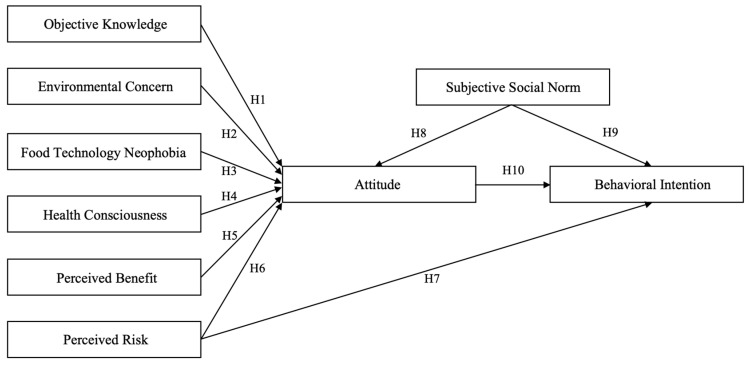
Framework developed based on empirical research and existing theories that we evaluated in the current study.

Based on our review of the literature and existing theories, we developed a hypothetical model to evaluate in the current study (Figure 1). The model consists of 10 explicit paths, each hypothesizing a direct effect of one variable on another variable. The model proposes that the following variables have a direct effect on attitude: objective knowledge, environmental concern, food technology neophobia, health consciousness, perceived benefit, perceived risk, and subjective social norm. It also proposes that attitude mediates the effect these variables have on behavioral intention toward irradiated ground beef and that attitude directly affects behavioral intention. In addition to their direct effect on attitude, the model proposes that perceived risk and subjective social norm directly affect behavioral intention.

## 3. Materials and Methods

### 3.1. Study Design

We used a cross-sectional survey research design. Such designs enable researchers to collect data at a single point in time [69] and provide a practical means to collect data from most populations efficiently [70]. Cross-sectional surveys are advantageous because they can be administered using various delivery modes [69,70], and they can measure many variables at one time [71]. We developed a survey instrument to collect data from participants.

### 3.2. Instrumentation

Participants first read the informed consent document and decided if they wanted to participate in the study. To participate, they needed to be at least 18 years old and eat ground beef. If they indicated they were younger than 18 and/or had never eaten ground beef, they were directed to the end of the survey. After answering those qualifying questions, participants read brief and objective information about food irradiation so as to not influence their responses. For those who were unfamiliar with irradiation, we wanted them to know that it is a food technology. Therefore, we asked them to consider the following information: Food irradiation is a technology used to treat food with radiation that comes from gamma rays, electron beams, or X-rays.

We measured participants’ objective knowledge of food irradiation using four items that we developed from information provided by the Food Irradiation Processing Alliance [72]. The four items were: (1) irradiated food generally has a longer shelf-life because spoilage organisms are reduced; (2) irradiated food sold in grocery stores is required to display the Radura symbol on its packaging; (3) food products are usually irradiated before packaging; and (4) the irradiation process kills harmful bacteria on food. The correct response to items 1, 2, and 4 was True, and the correct response to item 3 was False. Participants responded to each item using three response options: False (1), I don’t know (2), and True (3). We recoded ‘True’ responses as correct, and we recoded ‘I don’t know’ and ‘False’ responses as incorrect.

We measured participants’ environmental concern using four items from the New Ecological Paradigm (NEP) scale [73]. To determine which items to use, we conducted an exploratory factor analysis and selected the four items with the highest factor loadings on factor one. These four items were: (1) we are approaching the limit of the number of people the earth can support; (2) humans are severely abusing the environment; (3) the earth is like a spaceship with very limited room and resources; and (4) if things continue on their present course, we will soon experience a major ecological catastrophe. Respectively, these four items are items 1, 5, 6, and 15 in the NEP scale. Participants responded to the four items using the original NEP response scale—a 5-point Likert-type scale from Strongly disagree (1) to Strongly agree (5).

We measured participants’ food technology neophobia using four items from the Food Technology Neophobia Scale (FTNS) [45]. The four items were from the ‘New food technologies are unnecessary’ dimension of the scale. These four items were: (1) there are plenty of tasty foods around so we don’t need to use new food technologies to produce more; (2) the benefits of new food technologies are often grossly overstated; (3) new food technologies decrease the natural quality of food; and (4) there is no sense in trying out high-tech food products because the ones I eat are already good enough. The authors of [74] also used these four items from the FTNS to measure food technology neophobia. Participants responded to the four items using the original FTNS response scale—a 7-point Likert-type scale from Totally disagree (1) to Totally agree (7).

We measured participants’ health consciousness using three items from [75]. The three items were: (1) I am prepared to do anything that is good for health; (2) I often dwell on my health; and (3) I think that I take health into account a lot in my life. Participants responded to the items using a 6-point Likert scale from Strongly disagree (1) to Strongly agree (6). We chose to use a 6-point response scale because we wanted to force participants to choose whether they were more or less health conscious.

We measured participants’ perceived benefits of food irradiation using three items, each of which we developed from information provided by the Food Irradiation Processing Alliance [72]. The three items were: (1) the irradiation process can destroy harmful bacteria on food; (2) irradiation can make food safer to eat; and (3) the irradiation process preserves the taste and nutrients of food. Participants responded to the items using a 6-point Likert scale from Strongly disagree (1) to Strongly agree (6). We chose to use a 6-point response scale because we wanted to force participants to choose whether they perceived food irradiation as more or less beneficial.

We measured participants’ perceived risks of food irradiation using three items: one adapted from [11], one adapted from [76], and one adapted from [77]. The three items were: (1) eating irradiated food can increase the likelihood of contracting cancer; (2) eating irradiated food can damage human health; and (3) food irradiation can negatively impact the environment. Participants responded to the items using a 6-point Likert scale from Strongly disagree (1) to Strongly agree (6). We chose to use a 6-point response scale because we wanted to force participants to choose whether they perceived food irradiation as more or less risky.

We adopted Thyroff’s [78] definition of subjective social norm—“the perceived social pressure to consume or not to consume nanofoods” (p. 150) and adapted it to irradiated foods. We measured participants’ subjective social norms related to irradiated foods using three items—the first two we adapted from [78], and the third we developed. The three items were: (1) people who are important to me would approve of me consuming irradiated foods; (2) people who are important in my life think I should consume irradiated foods; and (3) people I know would consume irradiated foods. Participants responded to the three items using a 7-point Likert scale from Strongly Disagree (1) to Strongly Agree (7). We chose to use a 7-point response scale and include a middle (unsure) option because participants may have genuinely not known how people close to them felt about their consumption of irradiated foods.

We measured participants’ attitudes toward the use of food irradiation to treat food using three items we adapted from [79]. The first item, “Treating food with irradiation technology is ______,” included response options ranging from Extremely bad (1) to Extremely good (6). The second item, “Treating food with irradiation technology is ______,” included response options ranging from Extremely foolish (1) to Extremely wise (6). The third item, “I am ______ treating food with irradiation technology,” included response options ranging from Strongly against (1) to Strongly for (6). We chose to use a 6-point response scale because we wanted to force participants to choose whether their attitudes toward food irradiation were more positive or negative.

We measured participants’ behavioral intentions toward irradiated ground beef using three items we adapted from [80]. These items were: (1) I would purchase ground beef labeled ‘treated with irradiation’ in the grocery store; (2) I would eat irradiated ground beef; and (3) I would serve irradiated ground beef to my loved ones. Participants responded to the items using a 6-point Likert scale from Strongly disagree (1) to Strongly agree (6). We chose to use a 6-point response scale because we wanted to force participants to choose whether they were more or less likely to accept irradiated ground beef.

We also collected information about participants’ socio-demographic characteristics. The characteristics we measured were educational level completed, annual household income, marital status, status as a primary household grocery shopper, political ideology, racial/ethnic identity, age, state of residency, and gender identity.

### 3.3. Validity and Reliability

To establish the face and content validity of the instrument, we relied on an expert panel consisting of four social and behavioral science experts working in contexts of food and agriculture. We conducted a pilot study to examine the construct validity and internal consistency of measures. To collect pilot data, we used the Texas A & M University bulk email service and distributed one recruitment email to all students and employees at the College Station and Galveston campuses. Using pilot study data, we conducted exploratory factor analyses as an internal approach to determine the dimensionality of each scale. Using the final dataset, we conducted confirmatory factor analyses to confirm the factor structure, the results from which we report in Section 4.2. as part of the measurement model. To assess the internal consistency of each scale generating continuous data, we used Cronbach’s alpha (α). To assess the internal consistency of the objective knowledge scale, which generated dichotomous data, we used the Kuder–Richardson Formula 20 (KR-20). We report the reliability coefficients in Section 4.2.

### 3.4. Data Collection

We purchased a national quota sample from Qualtrics and collected complete responses from *N* = 1102 consumers. Qualtrics representatives ensured the data adhered to regional and gender quotas, meaning they sought to gather responses from a predetermined number of consumers who lived in the four U.S. regions and a predetermined number of consumers who identified as male or female. The predetermined numbers represented those expected nationally. Data collection took place from 8 December 2022 to 4 January 2023. Qualtrics representatives monitored the data collection process closely and performed various quality checks, one of which involved assessing responses to a quality check question included at the survey half point to ensure participants were responding meaningfully. We obtained approval from the Institutional Review Board of Texas A & M University to conduct the study (Study Number: 2022-0967M; Date of Approval: 11 July 2022).

### 3.5. Sample

The socio-demographic characteristics of the sample are presented in Table 1. Most participants identified as white (*f* = 850; 77.13%) and female (*f* = 568; 51.78%) and as the primary household food shopper (*f* = 927; 84.12%). Participants aged 65 to 74 accounted for the largest age group (*f* = 235; 18.62%), and participants who completed some college (*f* = 282; 25.59%) accounted for the largest education group. In addition, participants who earned between $35,000 and $74,999 annually accounted for the largest income group, and those who identified as moderate (*f* = 363; 32.94%) accounted for the largest political ideology group. Last, participants represented the four U.S. regions, and 44.01% were married (*f* = 485).

### 3.6. Data Analysis

We evaluated the model in Figure 1 using structural equation modeling (SEM) by means of Mplus version 8. First, we checked to see if the assumptions of SEM (i.e., linearity, no measurement error in the predictors, normality of residuals, homoscedasticity) were violated by the data using Stata/SE 17.0. To assess linearity, we used Residual versus Predictor Plots. To assess measurement error, we used Cronbach’s alpha and KR-20 as reliability estimates. To assess normality, we used the Density and QQ Plot, which suggested a close to normal distribution because the skewness was close to 0 (−0.41) and the kurtosis was close to 3 (4.06). To assess homoscedasticity, we used the Residual versus Fitted Plot. Based on our assessments, all assumptions were tenable. Second, we used descriptive statistics to examine variables’ means, standard deviations, and correlations. We also conducted single sample *t*-tests to compare each variable mean to the scale midpoint to understand if participants’ responses were statistically significantly different from a neutral or average position. Third, we assessed the measurement model by conducting a confirmatory factor analysis and evaluating construct validity (i.e., convergent validity, discriminant validity). Fourth, we analyzed the full structural model. We used five fit indices—the Root Mean Square Error of Approximation (RMSEA), the Weighted Root Mean Square Residual (WRMR), the Standardized Root Mean Square Residuals (SRMR), the Comparative-Fit Index (CFI), and the Tucker–Lewis Index (TLI)—to evaluate the measurement model and structural model.

### 3.7. Limitations

Limitations exist when using cross-sectional survey research designs, including common method bias, which may threaten the integrity of study results. Some sources of common method bias relate to the survey participants [81]. The data collected are self-reported, requiring participants to describe their own psychological and behavioral tendencies [66]. These data may be problematic, especially when the questions seek information “about socially-unacceptable behavior” because people tend to respond in a manner that they consider to be socially acceptable [69] (p. 369). We did our best to combat this source of common method bias by making it clear to participants, via the information sheet, that their response was anonymous. In addition, the external validity of the results may be compromised due to the differences in the data between those who respond to the survey and those who do not respond [69]. Qualtrics survey participants are registered to receive and complete surveys in exchange for compensation. Therefore, the results may be biased because they only reflect the opinions of this established participant pool. Other sources of common method bias relate to the survey instrument [81]. We strived to develop a sound instrument and mitigate sources of common method bias by using questions and items that were not complex or ambiguous and by ordering the survey questions in a way so as not to influence how participants answered subsequent questions. Still, there are other potential sources of common method bias in the study (e.g., measuring the independent and dependent variables at the same time in the same survey) that we were not able to control, and we acknowledge that those sources may impact the reliability of our study and the validity of our results [81]. Also limiting is that most survey participants were white and educated. Because of this, we are unable to generalize our results to a broader, diverse U.S. population.

## 4. Results

### 4.1. Descriptive Statistics

Participants had negative attitudes toward the use of irradiation to treat food (*M* = 3.16, *SD* = 1.17) (Table 2). We conducted a single sample *t*-test to compare the mean of attitude to the scale midpoint, which represents a neutral attitude toward food irradiation. Results suggest participants’ attitudes were significantly lower (*t*(1101) = −9.77, *p* < 0.001). Participants also had low objective knowledge of food irradiation (*M* = 2.00, *SD* = 1.34), and the results from the single sample *t*-test suggest their knowledge was significantly lower than average (*t*(1101) = −12.35, *p* < 0.001). In addition, participants perceived food irradiation to be risky (*M* = 3.65, *SD* = 1.13) and beneficial (*M* = 3.84, *SD* = 0.96), and both their risk and benefit perceptions were significantly higher compared to a neutral perspective (*t*(1101) = 4.37, *p* < 0.001; (*t*(1101) = 11.53, *p* < 0.001), respectively). They were somewhat unsure about how people close to them felt about their consumption of irradiated foods (*M* = 3.96, *SD* = 1.38), but their beliefs regarding norms were not significantly different from neutral (*t*(1101) = −0.98, *p* = 0.325). Moreover, participants were health conscious (*M* = 4.10, *SD* = 0.98)—more health conscious than average (*t*(1101) = 20.48, *p* < 0.001). They were also environmentally concerned (*M* = 3.83, *SD* = 0.95) and food technology neophobic (*M* = 4.38, *SD* = 1.30), both of which were also significantly different from neutral (*t*(1101) = 28.84, *p* < 0.001; (*t*(1101) = 9.72, *p* < 0.001), respectively).

Positive, statistically significant associations, substantial in strength, existed between attitudes and subjective social norms (*r* = 0.66, *p* < 0.001), attitudes and perceived benefits (*r* = 0.68, *p* < 0.001), and subjective social norms and perceived benefits (*r* = 0.62, *p* < 0.001) (Table 3). Positive and negative associations, statistically significant and of varying strengths, were identified between most of the variables.

### 4.2. Measurement Model

We considered the overall fit of the measurement model good. Table 4 includes the model fit indices meeting the most used criteria [83,84,85,86]. The RMSEA was 0.037, indicating an acceptable fit [87,88]. Because we included a categorical variable in the model, the WRMR was available in the data output and the SRMR was not. According to [85] and [86], the WRMR can be used as a good fit index for dichotomous data, such as those generated from the objective knowledge scale used in the current study, and it should be reported along with the CFI and TLI. The WRMR was 1.102—slightly above the good fit cutoff value of 1.0; however, higher cutoff values should be used when the measure includes fewer categories [85,86]. Therefore, a higher cutoff value may be necessary in this case, suggesting the WRMR value is acceptable. The CFI was 0.908 and the TLI was 0.891, both also indicating a good model fit [89,90].

We further determined the measurement model fit by evaluating diagnostic measures of convergent validity and discriminant validity to assess the overall construct validity. We evaluated factor loadings to assess convergent validity. Each item should have a factor loading of greater than 0.50 [91] and be statistically significant at the 0.05 alpha level [92]. All factor loadings were substantially greater than 0.50 except for those of two items measuring objective knowledge (Table 5). Items two and three of the objective knowledge scale had factor loadings of 0.48 and 0.49, respectively. However, loadings as low as 0.40 can be justified if acceptable values are obtained for other fit indices, like composite reliability (CR) [93]. In this case, the objective knowledge scale had a CR of 0.779, which far exceeds the acceptable cutoff value of 0.60 [91,94] and justifies the lower factor loadings. All standardized item loadings significantly loaded on their constructs (*p* < 0.001).

To further assess convergent validity, we evaluated the average variance extracted (AVE). Each construct should have an AVE value greater than 0.50 [93]. Each construct with the exception of objective knowledge and environmental concern had AVE values greater than 0.50. The AVE values for these two constructs were 0.488 and 0.456, respectively. However, similar to factor loadings, the convergent validity of the construct can still be considered adequate as long as the CR exceeds 0.60 [91,94]. Objective knowledge had a CR of 0.779 and environmental concern had a CR of 0.761, justifying the lower AVE values. All other constructs also had a CR greater than the recommended value of 0.60 [95]. In addition, all scales were internally consistent because Cronbach’s alpha coefficients were above 0.70, and the KR-20 coefficient was above 0.60 [96,97]. To assess discriminant validity, we evaluated the correlations between constructs. All correlations were less than the recommended value of 0.85 [93]. Thus, our results support the establishment of construct validity.

### 4.3. Structural Model

We considered the overall fit of the structural model good. Table 6 includes the model fit indices meeting the most used criteria [83,84]. The RMSEA was 0.018, indicating an acceptable fit [87,88]. The SRMR was 0.004, indicating a good fit [98]. The CFI was 0.999 and the TLI was 0.998—both larger than 0.90—also indicating a good fit [89,90]. The chi-square statistic was not significant (χ^2^(4, 1102) = 5.38, *p* = 0.251), further indicating good model fit [83,91].

The standardized indirect, direct, and total effects of the independent variables are reported in Table 7. The direct effects are the significant direct effects of particular variables on behavioral intention, and the indirect effects are the effects of significant mediating chains of a particular variable on behavioral intention [88]. Attitude toward food irradiation contributed the most to explaining the variance in participants’ behavioral intentions toward irradiated ground beef (β = 0.80, *p* < 0.001; total effect = 0.80). Subjective social norm also positively and directly affected behavioral intention (β = 0.10, *p* < 0.05) and had a mediating (indirect) effect on behavioral intention through attitude (β = 0.32, *p* < 0.001; total effect = 0.36). In addition, perceived benefit had a positive mediating (indirect) effect on behavioral intention through attitude (β = 0.35, *p* < 0.001; total effect = 0.28), as did objective knowledge though to a lesser extent (β = 0.07, *p* < 0.001; total effect = 0.06). Last, perceived risk had a negative direct effect on behavioral intention (β = −0.08, *p* < 0.01) as well as a mediating (indirect) affect through attitude (β = −0.22, *p* < 0.001; total effect = −0.26), and food technology neophobia had a small, negative mediating (indirect) effect on behavioral intention (β = −0.05, *p* < 0.05; total effect = −0.04). Environmental concern and health consciousness did not affect consumers’ behavioral intentions toward irradiated ground beef.

**Figure 2:**
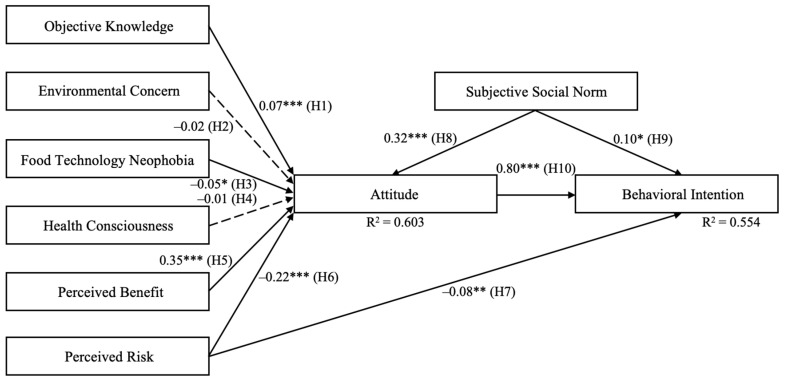
Results of evaluating the structural model explaining participants’ behavioral intentions toward irradiated ground beef.

Figure 2 includes the variance explained in the dependent variables and the standardized path coefficients. The model explained 60.3% of the variance in participants’ attitudes toward the use of irradiation to treat food and 55.4% of the variance in participants’ behavioral intentions toward irradiated ground beef. A solid arrow represents a significant path, and a dashed arrow represents an insignificant path. One asterisk indicates statistical significance at the 0.05 alpha level; two asterisks indicate statistical significance at the 0.01 alpha level; and three asterisks indicate statistical significance at the 0.001 alpha level.

Table 8 provides information regarding the conclusions for each hypothesis—either reject the hypothesis or fail to reject the null hypothesis.

## 5. Discussion

Teisl et al. [29] found U.S. consumers had negative attitudes toward food irradiation—a finding we confirmed in our study. These results, specific to U.S. consumers, are contrary to those of [67], who found Malaysian consumers had positive attitudes toward food irradiation, suggesting such attitudes vary by nationality. In addition, participants’ objective knowledge about food irradiation was low. These results are consistent with results from previous studies that also reported low knowledge levels among consumers, e.g., [16,18,24,25,26,27,28]. Therefore, it is reasonable to assume that despite food irradiation having been used in the agri-food sector for 60 years, communication efforts to increase consumers’ knowledge of the technology have been unsuccessful.

The theoretical model explained most of the variance in consumers’ attitudes toward food irradiation and behavioral intentions toward irradiated ground beef. Attitude [40,60,63] played the most critical role in consumers’ behavioral intentions toward irradiated ground beef, which was followed by subjective social norm [60,63,64]. Attitude also mediated the effect of subjective social norm [60,63,64,66], perceived benefit [23,24], perceived risk [23,24], objective knowledge [28,30,40], and food technology neophobia [44,49,50] on behavioral intention.

These results are like those of [63] who found that attitude and subjective social norm directly influenced consumers’ intent to accept and consume irradiated foods. However, they are unlike those of [63,64,65], as they analyzed data from 225 Minnesotans about purchase intentions toward irradiated beef patties and did not find a significant relationship between subjective norms and intentions to consume irradiated foods. It is possible that the importance of subjective social norms is increasing with time and playing a larger role in consumers’ decision-making processes when it comes to irradiated foods. Therefore, subjective social norm should be included as a key variable when investigating consumers’ behavioral intentions toward irradiated foods and when marketing irradiated foods to consumers.

To improve subjective social norms, food manufacturers, or communications and marketing specialists working for these companies, should consider designing visual aids that promote irradiated ground beef by emphasizing healthy families and social environments [66]. Doing so may improve subjective social norms by helping consumers envision people like them consuming such foods and, as a result, become more accepting of them [66]. Another science communication strategy that may improve subjective social norms is for communications and marketing specialists working for food manufacturers to develop messages from which the information source is another consumer. In other words, the message, whether it be delivered visually or in writing, should be delivered by a consumer. That way, consumers who receive and process the message may feel as though someone similar to them is accepting of these foods.

The effectiveness of these marketing strategies could be evaluated using in-person experimental research. For example, using a post-test-only control group research design, consumers in the treatment group could be exposed to an in-store display promoting irradiated ground beef that depicts a healthy family or a consumer who reflects the target audience in the respective area. Consumers in the control group (or groups) could be exposed to the same display without the image or the same display with a different image—perhaps one with ground beef or cattle. After consumers examine the display, they could respond to a post-test survey that seeks to understand their subjective social norms, attitudes, and intent to purchase the product. It is possible that different grocery stores in the same area and with similar customer bases (i.e., demographics, psychographics) could be used to recruit participants for the control and treatment groups.

As far as we know, our study is the first to provide evidence demonstrating the negative effect of food technology neophobia on consumers’ acceptance of irradiated food, despite other scholars discussing the relationship and presuming one exists, e.g., [44,48,49]. Therefore, we confirmed food technology neophobia is an important psychological factor researchers and practitioners must consider when attempting to understand, and change, consumers’ behaviors toward irradiated foods. Based on the food technology neophobia measurement used, consumers’ belief that new food technologies are unnecessary, specifically, had a negative effect on their behavioral intentions toward irradiated ground beef.

Our results suggest that the goal of science communication about food irradiation should be to increase consumers’ attitudes positively, which is best achieved by aiming to improve subjective social norms, increase perceived benefits, and decrease perceived risks [23,24]. Future research is needed to determine, more specifically, what those perceived risks are that most influence consumers’ decision making about irradiated foods (e.g., that they are radioactive) [1,9,11]. Although objective knowledge significantly influenced consumers’ attitudes toward food irradiation [29,30], its total effect was small. Thus, the goal of science communication efforts should not necessarily be to increase consumers’ knowledge of the technology because we found other psychological factors were more important.

One strategy that may increase perceived benefits and decrease perceived risks is communicating with consumers at the point-of-purchase using voluntary labels on food product packaging. Such messages should include information about the benefits of food irradiation, such as that it can increase the safety of consuming ground beef, that it preserves the nutrients in ground beef, and that it is an ecologically friendly process [99]. These particular messages may also correct common misperceptions of the technology [1,9,11,27,57]. The placement of voluntary labels that provide technology-related benefits should be considered (i.e., front of food package, back of food package), as should the context in which they are read (i.e., at the grocery store, at home), because consumers may respond differently under the various circumstances based on their psychological characteristics (e.g., subjective knowledge about nutrition, perceived importance of information, perceived credibility of information) [100]. Like the aforementioned experimental research study that could be conducted to determine if in-store displays depicting families and/or consumers improve consumers’ subjective social norms, a similar research design could be used to determine if voluntary labels including information about the benefits of food irradiation increase consumers’ perceived benefits and decrease their perceived risks.

Also important is the null effect of environmental concern on consumers’ attitudes toward food irradiation. Although consumers fear that the process of food irradiation pollutes the environment [18,19,39], their attitudes toward the technology are not affected by general concern for the environment. This could be because food irradiation is a processing method rather than a production method. Our study was the first to investigate environmental concern in the context of a food processing technology, but previous studies investigating environmental concern in the context of agricultural production technologies (i.e., genetic engineering) found it to influence consumers’ attitudes negatively, e.g., [18,34,35,36,37,38]. Future research should focus on determining if environmental concern does indeed affect consumers’ attitudes differently depending on when the technology is applied in the food supply chain.

Health consciousness also had a null effect on consumers’ attitudes toward food irradiation. Although consumers fear that irradiated foods might negatively impact their health [11,16,27], health consciousness did not affect acceptance. This finding came as a surprise because health-conscious consumers tend to prefer foods that they believe are natural and not processed [51]—two qualities they do not associate with irradiated foods [16,17,20,21]. Therefore, while communicating about the healthfulness and safety of irradiated foods is important to decrease perceived health risks, emphasizing other health-related information during communication is not necessary based on our results.

We recommend scholars evaluate the model to determine how well it explains consumers’ behavioral intentions toward other irradiated foods. Do the effects of predictor variables differ significantly when irradiation is used to treat fresh meat products other than ground beef? Do the effects of predictor variables differ significantly when irradiation is used to treat products of animal origin versus products of plant origin? Answers to these questions would help determine if the applicability of the model expands beyond the context of the current study. They would also help determine if the communication needs of consumers are different and, therefore, if marketing and communicating about irradiated foods needs to be different based on the product or if a one-size-fits-all approach would be effective.

A similar research recommendation is for scholars to evaluate the model to determine how well it explains consumers’ behavioral intentions toward foods produced or processed using other novel agri-food technologies, such as gene editing and nanotechnology. Such studies would respond to [18], who highlighted the need for using large sets of predictors to explain consumers’ acceptance of different agri-food technologies. Results from studies using the same model and measures would help us critically and accurately evaluate differences in consumers’ responses to agri-food technologies and understand how to leverage those differences, should they exist, when developing marketing and communication strategies. Evaluating the model further to determine its applicability in these various contexts would best be achieved using cross-sectional surveys with large, nationally representative consumer samples.

## Figures and Tables

**Table 1:** Socio-demographic characteristics of participants.

Characteristics	*N*	*f*	%
Age	1102		
18–24 years		114	10.34
25–34 years		172	15.61
35–44 years		184	16.70
45–54 years		127	11.52
55–64 years		181	16.42
65–74 years		235	21.32
75–84 years		73	6.62
85+ years		16	1.45
Gender Identity	1097		
Female		568	51.78
Male		504	45.94
Genderqueer (neither exclusively male nor female)		11	1.00
Female to male (FTM)/Transgender male/Trans man		7	0.06
Male to female (MTF)/Transgender female/Trans woman		4	0.36
Prefer not to respond		3	0.27
Highest Level of Education Completed	1102		
Some college		282	25.59
Bachelor’s degree		265	24.05
High school graduate		249	22.60
Associate degree (two-year college degree)		108	9.80
Master’s degree		105	9.53
Some high school		29	2.63
Trade/technical/vocational training		27	2.45
Professional degree		23	2.09
Doctoral degree		12	1.09
Other		2	0.18
Ethnicity	1102		
White		850	77.13
Black or African American		108	9.80
Hispanic		56	5.08
Asian		30	2.72
Multiracial		30	2.72
Prefer not to respond		13	1.18
American Indian or Alaska Native		7	0.64
Other		5	0.45
Native Hawaiian or other Pacific Islander		3	0.27
Income	1102		
Less than $10,000		68	6.17
$10,000–$24,999		163	14.79
$25,000–$34,999		163	14.79
$35,000–$74,999		356	32.30
$75,000–$99,999		130	11.80
$100,000–$149,999		116	10.53
$150,000–$249,999		51	4.63
$250,000 or more		15	1.36
Prefer not to respond		40	3.63
Political Ideology	1102		
Very liberal		95	8.62
Liberal		128	11.62
Somewhat liberal		112	10.16
Moderate		363	32.94
Somewhat conservative		143	12.98
Conservative		157	14.25
Very conservative		104	9.44
Marital Status	1102		
Married		485	44.01
Single, never married		352	31.94
Divorced		173	15.70
Widowed		70	6.35
Separated		22	2.00
Primary Household Grocery Shopper	1102		
Yes		927	84.12
No		175	15.88
U.S. Region	1102		
South		432	39.20
West		241	21.87
Midwest		241	21.87
Northeast		180	16.33

**Table 2:** Means and standard deviations of the independent variables (*N* = 1102).

Variable	*M*	*SD*	*t*-Value	*p*-Value
Food technology neophobia (7-point response scale)	4.38	1.30	9.72	<0.001
Health consciousness (6-point response scale)	4.10	0.98	20.48	<0.001
Subjective social norm (7-point response scale)	3.96	1.38	−0.98	0.325
Perceived benefit (6-point response scale)	3.84	0.96	11.53	<0.001
Environmental concern (5-point response scale)	3.83	0.95	28.84	<0.001
Perceived risk (6-point response scale)	3.65	1.13	4.37	<0.001
Attitude (6-point response scale)	3.16	1.17	−9.77	<0.001
Objective knowledge *	2.00	1.34	−12.35	<0.001

Note: * indicates the variable mean was derived from summing the number of correct answers (four questions total).

**Table 3:** Pearson product–moment correlations between independent variables (*N* = 1102).

Variable	1	2	3	4	5	6	7	8
Knowledge (1)	1							
Environmental concern (2)	0.06 *	1						
Food technology neophobia (3)	−0.13 ***	0.04	1					
Health consciousness (4)	0.05	0.14 ***	0.18 ***	1				
Perceived benefit (5)	0.44 ***	0.04	−0.24 ***	0.01	1			
Perceived risk (6)	−0.21 ***	0.11 ***	0.45 ***	0.22 ***	−0.42 ***	1		
Subjective social norm (7)	0.34 ***	0.01	−0.23 ***	0.06 *	0.62 ***	−0.41 ***	1	
Attitude (8)	0.37 ***	−0.03	−0.30 ***	−0.05	0.68 ***	−0.53 ***	0.66 ***	1

Note: * *p* < 0.05, *** *p* < 0.001. 0.01–0.09 = Negligible association; 0.10–0.29 = Low association; 0.30–0.49 = Moderate association; 0.50–0.69 = Substantial association; 0.70 or higher = Very strong association [82].

**Table 4:** Model fit of the measurement model.

Criteria	Thresholds for Acceptable Fit	Measurement Model
RMSEA	<0.05–0.08	0.037
WRMR	<1.00	1.102
CFI	>0.90	0.908
TLI	>0.90	0.891

**Table 5:** Measurement model assessment of constructs and associated items.

Constructs and Items	Factor Loading	Reliability Coefficient	CR	AVE
Objective knowledge		KR-20 = 0.63	0.779	0.488
Item 1	0.824			
Item 2	0.476			
Item 3	0.493			
Item 4	0.895			
Environmental concern		α = 0.77	0.761	0.456
Item 1	0.616			
Item 2	0.644			
Item 3	0.615			
Item 4	0.782			
Food technology neophobia		α = 0.81	0.806	0.516
Item 1	0.628			
Item 2	0.629			
Item 3	0.899			
Item 4	0.681			
Health consciousness		α = 0.77	0.772	0.531
Item 1	0.668			
Item 2	0.749			
Item 3	0.766			
Perceived benefit		α = 0.80	0.806	0.589
Item 1	0.564			
Item 2	0.873			
Item 3	0.829			
Perceived risk		α = 0.88	0.884	0.720
Item 1	0.725			
Item 2	0.916			
Item 3	0.892			
Subjective social norm		α = 0.85	0.850	0.655
Item 1	0.871			
Item 2	0.836			
Item 3	0.712			
Attitude		α = 0.93	0.935	0.826
Item 1	0.884			
Item 2	0.908			
Item 3	0.935			
Behavioral intention		α = 0.96	0.955	0.877
Item 1	0.926			
Item 2	0.934			
Item 3	0.949			

Note: Item numbers correspond with those listed in Section 3.2.

**Table 6:** Model fit of the structural model.

Criteria	Thresholds for Acceptable Fit	Structural Model
χ^2^ (df)	--	5.378
χ^2^/df	<3.00–5.00	1.345
RMSEA	<0.05–0.08	0.037
SRMR	<0.08	0.004
CFI	>0.90	0.999
TLI	>0.90	0.998

**Table 7:** Standardized indirect, direct, and total effects of variables on behavioral intention toward irradiated ground beef (*N* = 1102).

Independent Variable	Indirect Effect	Direct Effect	Total Effect
Attitude	--	0.80	0.80
Subjective social norm	0.26	0.10	0.36
Perceived benefit	0.28	--	0.28
Perceived risk	−0.18	−0.08	−0.26
Objective knowledge	0.06	--	0.06
Food technology neophobia	−0.04	--	−0.04

**Table 8:** Conclusions for hypotheses.

	Hypothesis	Conclusion
H1	Environmental concern has a significant negative effect on attitude toward food irradiation.	Not supported
H2	Food technology neophobia has a significant negative effect on attitude toward food irradiation.	Supported
H3	Health consciousness has a significant negative effect on attitude toward food irradiation.	Not supported
H4	Objective knowledge has a significant positive effect on attitude toward food irradiation.	Supported
H5	Perceived benefit has a significant positive effect on attitude toward food irradiation.	Supported
H6	Perceived risk has a significant negative effect on attitude toward food irradiation.	Supported
H7	Perceived risk has a significant negative effect on behavioral intention toward irradiated ground beef.	Supported
H8	Subjective social norm has a significant positive effect on attitude toward food irradiation.	Supported
H9	Subjective social norm has a significant positive effect on behavioral intention toward irradiated ground beef.	Supported
H10	Attitude has a significant positive effect on behavioral intention toward irradiated ground beef.	Supported

## Data Availability

The data used to support the findings of this study can be made available by the corresponding author upon request.
